# Bioactive secondary metabolites from marine *Actinomyces* sp. AW6 with an evaluation of ADME-related physicochemical properties

**DOI:** 10.1007/s00203-022-03092-5

**Published:** 2022-08-01

**Authors:** Mohamed A. Agour, Ahmed A. Hamed, Mosad A. Ghareeb, Eman A. A. Abdel-Hamid, Mohamed K. Ibrahim

**Affiliations:** 1 Botany Department, Faculty of Science, Zagazig University, Zagazig, Egypt; 2 Microbial Chemistry Department, National Research Centre, 33 El-Buhouth Street, Dokki, Giza, 12622 Egypt; 3 Medicinal Chemistry Department, Theodor Bilharz Research Institute, Kornaish El Nile, Warrak El-Hadar, Imbaba, Giza, 12411 Egypt; 4 Central Laboratory for Aquaculture Research, Abbassa, Abu-Hammad, Sharqia, Egypt; 5 Microbiology Department, Faculty of Science, Ain Shams University, Cairo, 11566 Egypt

**Keywords:** *Actinomyces* sp. AW6, Secondary metabolites, Antioxidant, Antimicrobial, Anti-Gyr activity, ADME

## Abstract

This study was designed to evaluate the antimicrobial, antioxidant, and cytotoxic potentials of the marine actinomycetes spp. isolated from the Red Sea water, Hurghada, Egypt. Out of 80 actinomycetes isolates, one isolate AW6 was selected based on its antioxidant activity (IC_50_ about 5.24 µg/mL which scavenged 91% of formed DPPH free radicals) and antimicrobial potential against *E. coli*, *S. aureus*, *B. subtilis,* and *P. aeruginosa, A. niger,* and *C. albicans*. The strain was identified based on phenotypic and genotypic analysis, and deposited in the GenBank with accession number OK090864.1. Cultivation of the selected strain on rice, chromatographic purification, and structural elucidation led to the isolation of two compounds **C1**: umbelliferone, and **C2:** 1-methoxy-3-methyl-8-hydroxy-anthraquinone. The antimicrobial activity of the obtained compounds showed that C1 and C2 have low antibacterial activity toward *S. aureus* and *E. coli* with no pronounced activity toward *P. aeruginosa*, *C. albicans*, and *A. niger.* Additionally, the antioxidant activity of **C1** and **C2** revealed that **C2** has a good antioxidant activity, with DPPH scavenging activity reaching (55.25%), followed by C**1** (30.20%). Moreover, both compounds displayed anti-Gyr-B enzyme activity with IC_50_ value of (3.79 ± 0.21 µM) for **C1**, and (IC_50_ = 13 ± 0.71 µM) for **C2**. The ADME-related physicochemical properties of the obtained compound were predicted using SwissADME web tools and the ProToxii webserver was used to estimate in silico toxicity.

**Supplementary Information:**

The online version contains supplementary material available at 10.1007/s00203-022-03092-5.

## Introduction

Actinomycetes are the most abundant category of microorganisms in nature, primarily found in soil and marine water (Abdel-Aziz et al. 2018). They have produced a large number of essential bioactive chemicals with great economic value and are still constantly tested for the production of new bioactive molecules. Actinomycetes contain around two-thirds of naturally occurring antibiotics, including several medically significant compounds (Palazzotto et al. 2019). Almost 80% of the world’s antibiotics are known to be derived from Actinomycetes, primarily the species Streptomyces and Micromonospora (van der Meij et al. 2017). Most antibiotics are no longer effective against the majority of bacteria-caused infections (Ghareeb et al. 2015; Elkhouly et al. 2021a). *Staphylococcus aureus* is a well-known organism that causes illnesses such as pneumonia and recently has gained resistance to the majority of antibiotic groups (Tong et al. 2015; Hamed et al. 2020a, b; El-Shazly et al. 2021). During this time, vancomycin has been the therapeutic antibiotic against Methicillin-resistant *Staphylococcus aureus* (MRSA), but unfortunately, Vancomycin-resistant strains have emerged clinically (Gardete and Tomasz 2014). Vancomycin-resistant *S. aureus* (VRSA) is considered a critical problem to doctors not only because of its vancomycin and methicillin resistance but also due to resistance to a variety of other antibiotics, including aminoglycosides, fluoroquinolones, and macrolides (Kest and Kaushik 2019). Certain unfavorable side effects and the spread of diseases associated with this novel antimicrobial medication resistance highlight the necessity for the development of alternative newer antimicrobial medicines with effectiveness against Gram-positive bacteria (Mohammed et al. 2019; Elkhouly et al. 2021b). Furthermore, Gram-negative antibiotic-resistant opportunistic bacteria with a multi-drug resistance threaten patients in hospitals and communities as they possess a widespread resistance to the first, second, and third generations of penicillin and cephalosporin (Mohammed et al. 2019). These bacteria, such as *Pseudomonas aeruginosa*, are widespread organisms found in the environment that operate as opportunistic pathogens in clinical circumstances where the patient’s defense system is impaired (Moradali et al. 2017**)**. As a result of this phenomenon, many strains of bacteria have developed antibiotic resistance, and in many cases, multi-resistant, to these known therapeutic medicines, and to address this issue, a new antibiotic with a novel mechanism of action is required (Ventola 2015).

The present study is carried out to explore the role of Actinomycetes compounds as antioxidant and antimicrobial agents against different types of pathogens including *P. aeruginosa*, *S. aureus*, *E. coli*, *A. niger*, and *C. albicans.* The anti-Gyr activity of the isolated compounds was also investigated. Furthermore, the ADME-related physicochemical properties of the obtained compound were predicted using SwissADME web tools and the ProToxii webserver was used to estimate in silico toxicity.

## Materials and methods

### Collection of sediment samples

In June 2018, eight marine samples were obtained from the Red Sea at depths 10, 15. 32 and 40 m using a sterile core sampler. The sediment samples were collected and serially-numbered before being transported to the Microbiology laboratory and kept at 4 °C at the National Research Centre.

### Isolation of actinomycetes from sediment samples

The isolation of marine actinomycetes was performed using a starch nitrate agar medium containing 50% salt water and the following ingredients: (g/L): 20 starch; 0.5 K_2_HPO_4_; 1 KNO_3_; 0.5 MgSO_4_ 7H_2_O; 0.01 FeSO_4_; and 15 agar. Before sterilization, the pH of the medium was adjusted to 7, and Mycostatin (500 L/L) was added afterward. 1 g of sediment was taken from each sample and mixed separately with 9 mL of sterile saltwater. In 100 mL of sterile starch nitrate agar medium, five milliliters of the suspension were added. Each sterilized plate received approximately 20 mL of medium with no air bubbles. The inoculated plates were incubated for 7 days at 30 °C. After incubation, the actinomycete colonies were picked and purified using the streaking method. The purified strains were preserved on starch nitrate slants and 25% glycerol stocks at − 20 °C (Burhamzah et al. 2016).

### Small-scale fermentation and screening of antimicrobial activity

Each isolate was grown on rice substrate, extracted with ethyl acetate, and evaporated to test its antimicrobial activity. The agar well diffusion method was used to test the antimicrobial activity of crude actinomycetes extracts. Sterile Mueller Hinton agar plates were swabbed separately with the pathogens as test microbes including Gram-positive bacteria; *Bacillus subtilis* (ATCC66), *Staphylococcus aureus* (ATCC6538-P), and Methicillin-resistant *Staphylococcus aureus* (MRSA) (ATCC25923); Gram-negative bacteria *Escherichia coli* (ATCC14169), *Pseudomonas aeruginosa (*ATCC 27853), *Klebsiella pneumoniae, Salmonella typhi*, and *Salmonella enterica*; yeasts *Candida albicans* (ATCC10231), and (ATCC9080) and fungus *Aspergillus niger* (NRRL A-326). In each plate, 6 mm diameter wells were drilled with a sterile cork borer and approximately 20 µL of each extract was put into each well against each of the test organisms. After that, plates were incubated for 24 h at 37 °C. The diameter of the inhibition zones was used to determine antimicrobial activity. Only isolates with broad-spectrum activity were chosen for further study (Waithaka et al. 2017). Antimicrobial activity was observed after 24 h of incubation at 37 °C for bacteria and 48 h of incubation at 25 °C for fungus, and inhibition zones were reported as diameters (mm).

### Identification of the most potent isolate

#### Phenotypic identification

Morphological methods, including macroscopic and microscopic ones, were used to characterize the potent isolate up to the genus level. Through oil immersion (100X), the mycelium structure, color, and arrangement of conidiophores were observed. The studying of spore-bearing hyphae, spore chain structure, spore color, aerial mass color, and substrate mycelia color were studied according to Bergey (1989) and the International Streptomyces Project (ISP). Additionally, morphological and cultural characteristics of the potent isolate AW6 were studied in different media following the instructions given by the International *Streptomyces* Project (ISP) (Singh et al. 2012). The biochemical tests were performed by assessing the oxidase enzyme ability, indole test, methyl red test, citrate utilization ability, carbohydrate fermentation, and hydrogen sulfide production nitrate reduction (Pradhan and Tamang 2019).

#### Genotypic identification

Based on the antimicrobial activity of the crude extracts, one isolate coded AW6 was genetically identified by sequencing the isolate’s 16S rRNA gene. The Qiagen DNeasy Blood & Tissue Kit (Thermo Fisher Scientific, Waltham, USA) was used to extract the DNA, which was done according to the manufacturer’s recommendations. Two universal primers were used in the PCR amplification (27F5′-AGAGTTTGATCCTGGCTCAG-3′; 1492R 5′-GGTTACCTTGTTACGACTT-3′). The final volume of the PCR amplification reaction was as follows: 50 μL (5 μL of 10 × Dream Taq Green PCR buffer, 2 μL of each 10 μmol dm^−3^ primers, 5 μL of 2 mmol dm^−3^ dNTP, 0.3 μL Taq DNA polymerase, and 0.5 μL of template DNA). The PCR reaction ran under the following conditions: 94 °C for 45 s, 55 °C for 60 s, and 72 °C for 60 s. The purified product was sequenced at Macrogen Company, South Korea. The similarity and homology of the 16S rDNA sequences were investigated by comparing the obtained sequences with similar known sequences in the NCBI database using online BLAST alignment search tools (http://www.ncbi.nlm.nih.gov/BLAST). MEGA-X software was used to create the phylogenetic tree (Kumar et al. 2016).

### Fermentation and production of bioactive compounds

Rice in the solid state was used as a fermentation medium; the selected isolate was grown into a 250 mL Erlenmeyer flask containing 50 mL starch nitrate broth (pH 7) at 30 °C for 7 days under shaking conditions. After incubation, the starch broth culture medium was used as a seed culture to inoculate the rice media for large-scale fermentation (Hamed et al. 2022).

### Extraction of bioactive compounds

Extraction of the bioactive metabolites from the fermented rice medium inoculated with *Actinomyces* sp. AW6 was carried out using an equal volume of ethyl acetate after shaking for one hour for complete extraction (Hamed et al. 2022). The ethyl acetate was separated and evaporated at 40 °C to obtain 13 g of the dry extract.

### Purification of bioactive secondary metabolites

Purification and structure elucidation were carried out initially using flash column chromatography. 10 g from the obtained crude extract was applied on a 7 cm diameter column filled with normal phase silica. The ratio of 20:1 adsorbent (silica gel) to solute (crude extract). A total of 100 fractions of 10 mL each were collected and analyzed using thin-layer chromatography (TLC) to identify the fractions with compounds of interest. The most potent fraction was further purified using the size-exclusion chromatography technique and performed using Sephadex LH-20 sub-column. The separation was based on molecular weight. The purified compounds were subjected to different spectroscopic analyses such as NMR and ESI for structure elucidation.

### Biological activity evaluation

#### Antimicrobial activity

The antimicrobial activity measurement of *Actinomyces sp.* AW6 compounds was carried out using an antimicrobial assay and MIC as described by Hamed et al. (2020a, b), Alhadrami et al. (2021), and Qader et al. (2021). All test pathogens were obtained from the Culture Collection Center (Microbial Chemistry Department and National Research Centre, NRC), Egypt.

#### Determination of free radical scavenging activity (RSA)

The free radical scavenging activity (RSA) was measured by the decoloration of an ethanolic solution of DPPH radical spectrophotometrically at 517 nm following Brand-Williams et al (1995) technique. The scavenging activity was calculated as follows:

$${\text{Scavenging ability }}\left( \% \right) \, = \, \left( {A_{{517{\text{ of control}}}} - \, {{A_{{517{\text{ of sample}}}} } \mathord{\left/ {\vphantom {{A_{{517{\text{ of sample}}}} } {A_{{517{\text{ of control}}}} }}} \right. \kern-\nulldelimiterspace} {A_{{517{\text{ of control}}}} }}} \right) \, \times 100.$$

### In vitro DNA gyrase-B inhibition

The DNA gyrase-B and ParE inhibitory activities were determined using the Inspiralis assay kit (Inspiralis^®^, London, UK) on streptavidin-coated 96-well microtiter plates (Thermo Scientific, Hamburg, Germany), according to the manufacturer’s protocol (Durcik et al. 2018). The experiment measures the capacity of the separated drugs to inhibit the ATPase activity of both the gyrase-B and ParE subunits.

### In silico predictions ADME-related physicochemical properties

The ADME-related physicochemical properties of the obtained compound were predicted using SwissADME web tools (Daina et al. 2017).

### In silico toxicity prediction

The in silico prediction of toxicity for compounds was performed via ProTox ii web server as previously reported **(**Banerjee et al. 2018).

## Results and discussion

### Isolation of endophytic actinobacteria

Using serial dilution technique, several actinobacterial strains were isolated from collected marine samples, i.e., algal homogenate, marine sediment, or marine water. Several studies have isolated many actinomycetes isolates from marine samples, and the isolation was depended on visualization of actinomycetes colonies on specific media for actinomycetes (Hamed et al. 2022), Table 1 summarizes the number of isolates and their sources.

**Table 1:** Distribution and percent of actinobacteria isolated from different marine localities

Location	Number of isolates	Percentage incidence (%)
Seawater Hurghada	28	35
RasSedr sediments	24	30
AinSokhna sediment	20	25
Marine algae	8	10
Total isolate	80	100

### Antimicrobial screening for isolated endophytic actinobacteria

Eighty isolates were selected based on morphological examination as actinomycetes and screened biologically for their antimicrobial activity. Based on the antimicrobial activity of the tested strains, the isolate AW6 has been selected for further investigation Table 2 shows the antimicrobial activity of some selected actinomycetes. Actinomycetes species have been mentioned in several reports as a source of bioactive secondary metabolites such as cytotoxic compounds (Yoo et al. 2002; Thangapandian et al. 2007) and antimicrobial compounds (Kumari et al. 2006; Rizk et al. 2007) that have the potential to control a wide range of pathogens. Millions of microorganisms, including indigenous actinomycetes, live in the marine environment and play an important role in the mineralization of complex organic matter, degradation of dead plants, plankton, and animals, removal of pollutants and toxicants, and production of primary and secondary metabolites (Genilloud et al. 2011). There are many unique and different types of actinomycetes in the maritime environment (Lam 2007). The isolation of novel secondary metabolites from distinct populations of actinomycetes from the marine environment suggests that these microorganisms represent an important new resource for marine and microbial natural product research (Abdel-Mageed et al. 2010; Asolkar et al. 2010; Li et al. 2011; Roh et al. 2011; Sousa et al. 2012; Jensen et al. 2005). Continuing attempts to study the variety of marine actinomycetes and how their development in the marine environment has influenced the generation of bioactive secondary metabolites have provided insights into how these microorganisms might be exploited to manufacture bioactive chemicals (Zhang et al. 2011).

**Table 2:** Antimicrobial activity of actinobacterial crude extracts

Extracts	Antibacterial activity (clear zone, mm)						Antifungal activity (clear zone, mm)		
	*Gram −ve*			Gram + ve					
	*E. coli*	*P. aeruginosa*	*K. pneumonia*	MRSA	*B. subtilis*	*S. aureus*	*C. albicans* I	*C. albicans* II	*A*. niger
AW2	NA	10.8 ± 0.23	NA	NA	11.9 ± 0.45	9.9 ± 0.2	NA	10.9 ± 0.23	NA
AW 6	17.6 ± 0.24	23.8 ± 0.34	21.8 ± 0.12	17.8 ± 0.2	25.7 ± 0.3	22.3 ± 0.4	17.6 ± 0.24	23.7 ± 0.34	9.2 ± 0.10
AR3	NA	13.2 ± 0.52	NA	NA	NA	NA	10.3 ± 0.13	13.2 ± 0.52	NA
AR10	9.7 ± 0.13	NA	NA	NA	9.1 ± 0.18	NA	9.9 ± 0.13	NA	NA
B 9	NA	NA	NA	NA		NA	12.7 ± 0.23	11.3 ± 0.43	NA
B 10	NA	NA	NA	NA	NA	NA	NA	NA	NA
B 11	NA	NA	NA	NA	NA	NA	NA	NA	NA
Strep:	17.2 ± 0.10	24. 6 ± 0.20	20.0 ± 0.31	23.2 ± 0.12	25.3 ± 0.19	23.8 ± 0.31	–	–	–
Amp:	–	–	–	–	–	–	22.9 ± 0.12	25.3 ± 0.19	9.6 ± 0.31

Each value in the table is the mean ± standard deviation of three trials

*NA* not active, *Strep.* Streptomycin, *Amp* amphotericin B

### Identification of the most potent strain AW6

#### Phenotypic identification

The morphological studies of the AW6 strain showed that the spore chain is rectiflexibiles with a smooth spore surface (Supplementary 1). The cultural properties of the AW6 strain after cultivation on different ISP media were summarized in Supplementary (2). Additionally, the physiological and biochemical characteristics for the selected isolate AW6 include nitrate reduction, catalase, methyl red (MR), indole production, Voges–Proskauer (V–P), starch hydrolysis, hydrogen sulfide production, and gelatin hydrolysis were also assessed and represented in Supplementary (3). The production of acid from carbohydrates was investigated with *Actinomyces* fermentation broth (BBL) as the basal medium. xylose, Arabinose, mannose, glucose, lactose, cellobiose, glycerol, raffinose, glycogen, salicin, mannitol, rhamnose, starch, inositol, and sorbitol were tested. The used tests in the biochemical study are performed as described in the report of the International Subgroup on the Taxonomy of Microaerophilic Actinomycetes (Slack 1968).

#### Genotypic identification

**Fig. 1:**
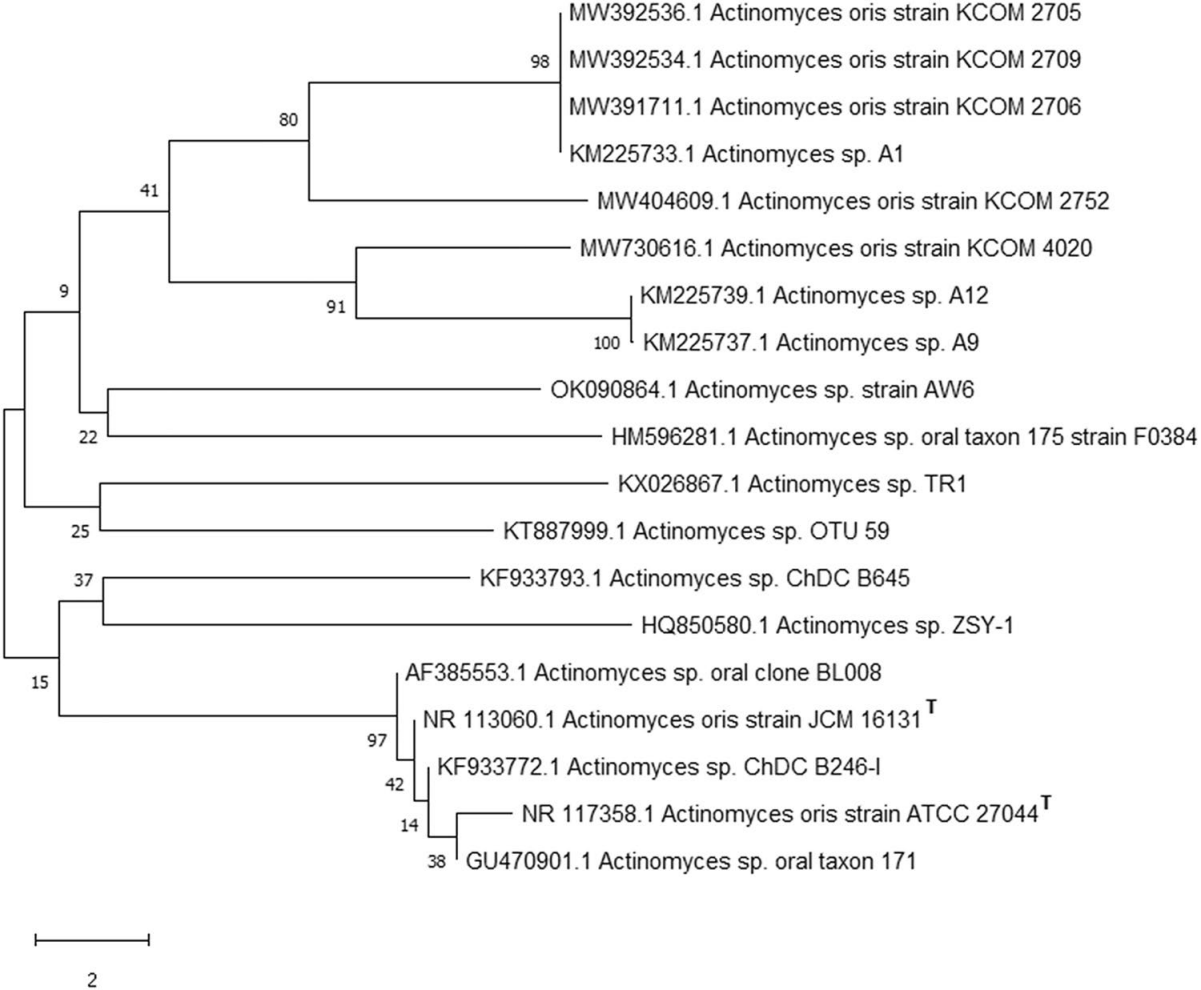
Phylogenetic tree of the *Actinomyces* sp. AW6

The 16S rRNA gene was extracted, amplified, sequenced, and aligned against known sequences deposited in the GeneBank database via the Basic Local Alignment Search Tool (BLAST) tool to measure the similarity score and calculate the statistical significance of the matches (http://www.blast.ncbi.nlm.nih.gov/Blast). The obtained result showed a very close similarity of the obtained sequence with 100% homology of the isolate AW6 with *Actinomyces* sp. Based on the analysis of the DNA sequence and the morphological characteristics of the AW6 isolate was identified as *Actinomyces* sp. AW6 and deposited in GenBank with accession no. OK090864.1. The maximum likelihood approach and the Tamura–Nei model were used to infer the evolutionary history (Fig. 1). The graph displays the tree with the highest log likelihood (− 17,134.78). The proportion of trees with the relevant taxa grouped is presented beside the branches. The tree is shown to scale, and branch lengths are quantified in terms of the number of substitutions per site. This study included 14 nucleotide sequences. The final dataset had a total of 1531 locations. MEGA-X was used to perform evolutionary analysis.

### Large-scale production and purification of bioactive compounds

The strain *Actinomyces sp.* AW6 was cultivated on rice medium and extracted with ethyl acetate. The ethyl acetate evaporated using a rotary evaporator till complete evaporation of ethyl acetate. The extract (13 g) was primarily fractionated using Flash column chromatography into 100 fractions. Based on the chemical screening using TLC and biological screening, the most active fractions were collected together further and purified using Sephadex LH-20 column with a gradient mobile phase DCM: Methanol. The TLC chromatogram showed that fraction no. (8) contains the peaks of interest. The structural elucidation of the isolated compounds was performed using nuclear magnetic resonance (NMR) spectroscopy.

**Fig. 2:**
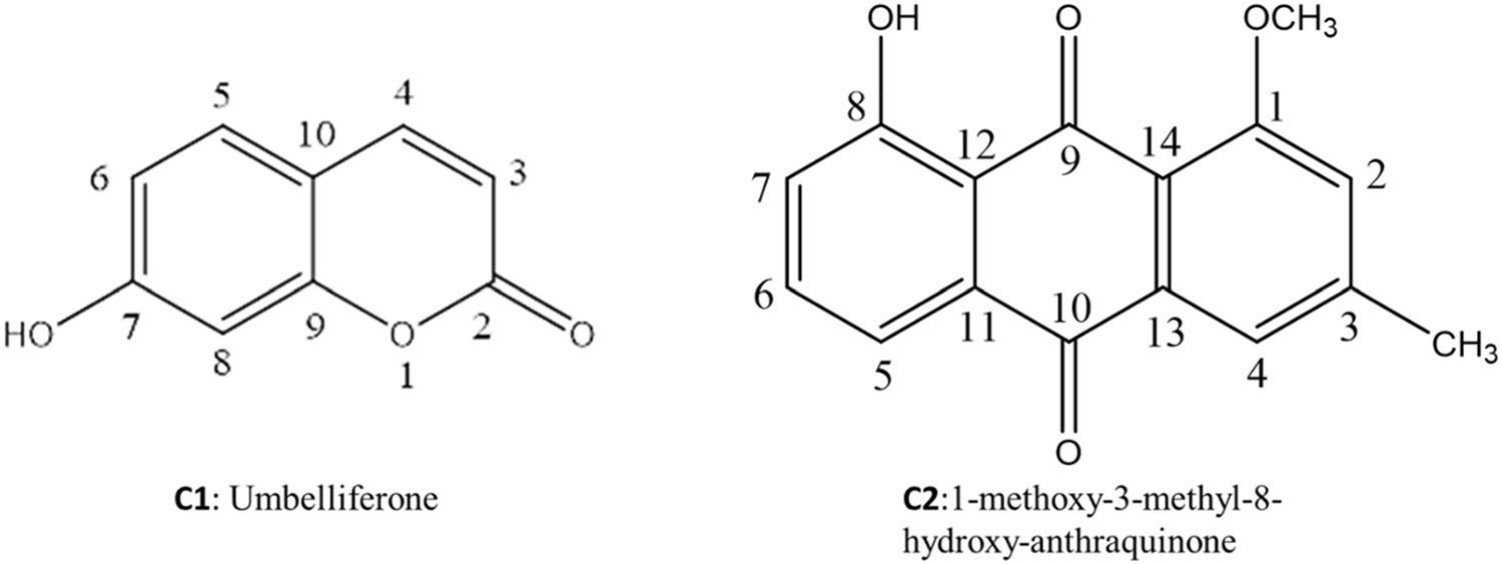
Isolated compounds from *Actinomyces sp.* AW6

**Compound (1)** was obtained as white needles. ^1^H NMR spectra showed characteristic signals for ABX system of three aromatic protons at δ_H_ (ppm): 6.72 (IH, dd, *J* = 8.0, 2.1 Hz, H-6), 7.28 (1H, d, *J* = 8.0 Hz, H-5), and 6.75 (lH, d, *J* = 2.1 Hz, H-8) indicating to the presence of 1,2,4-trisubstituted benzene ring. Moreover, 1,2-disubstituted olefinic protons were resonated at δ_H_ (ppm): 6.19 (1H, d, *J* = 9.5 Hz, H-3)*,* and 7.61 (1H, d, *J* = 9.5 Hz, H-4) indicating the presence of α,β-unsaturated ketone of a coumarin ring. ^13^C NMR spectra revealed the presence of characteristic signals for aromatic, olefinic and carbonyl carbons at δ_C_ (ppm): 162.0 (C-2), 113.7 (C-3), 145.0 (C-4), 129.89 (C-5), 112.1 (C-6), 160.97 (C-7), 102.94 (C-8), 156.2 (C-9), and 111.69 (C-10). Therefore, the compound could be identified as umbelliferone based on its chromatographic properties, proton and carbon spectra, and available reported data (Fig. 2) (Wagh et al. 2021).

**Compound (2)** was obtained as a pale orange powder. ^1^H NMR spectra showed characteristic signals for aromatic protons were resonated at δ_H_ (ppm): 7.10 (1H, s, H-2), 7.34 (1H, s, H-4), 7.33 (1H, d, *J* = 6.8, H-5), 7.74 (1H, dd, *J* = 8.2 and 6.8, H-6), and 7.51 (1H, d, *J* = 8.2, H-7) indicating the presence of quinone nucleus. Additionally, methyl and methoxy groups were resonated at δ_H_ (ppm): 2.38 (3H, s, –CH_3_) and 3.71 (3H, s, –OCH_3_). ^13^C NMR spectra revealed the presence of characteristic signals for aromatic, carbonyls, aromatic methyl, and methoxy carbons at δ_C_ (ppm): 157.0 (C-1), 115.7 (C-2), 118.9 (C-3), 122.36 (C-4), 118.7 (C-5), 137.1 (C-6), 122.9 (C-7), 160.93 (C-8), 185.0 (C-9), 182.5 (C-10), 132.6 (C-11), 113.9 (C-12), 138.9 (C-13), 128.2 (C-14), 56.1 (–OCH_3_-1), and 21.2 (–CH_3_-3) supporting the presence of anthraquinone skeleton. Based on its chromatographic properties, proton and carbon spectra, and available reported data, the compound could be identified as 1-methoxy-3-methyl-8-hydroxy-anthraquinone (Fig. 2) (Kumar et al. 2017).

### Biological evaluation

#### Antimicrobial activity

The antimicrobial activity testing of compounds C1 and C2 using MTP assay revealed that compounds C1 and C2 have low antibacterial activity toward *S. aureus* and *E. coli.* While no pronounced activity has been detected toward the rest microbes including *P. aeruginosa*, *C. albicans* and *A. niger.* The minimum inhibitory concentration of the two compounds has been recorded in Table 3.

**Table 3:** MIC of the isolated compounds (C1 and C2), the antibiotic streptomycin and the antifungal fluconazole

Compounds	MIC (µg/mL)				
	*S. aureus*	*E. coli*	*P. aeruginosa*	*C. albicans*	*A. niger*
Compound 1	125	250	–	–	–
Compound 2	125	125	–	–	–
Streptomycin	< 0.78	6.25	25	–	–
Amphotericin B	–	–	–	0.50	5.00

#### Antioxidant activity

The antioxidant activity of compounds **(C1** and **C2)** based on 2,2-diphenyl-1-picrylhydrazyl radical (DPPH assay; 200 µg/mL) revealed that 1-methoxy-3-methyl-8-hydroxy-anthraquinone (**C2**) is the most antioxidant agent, showed maximum DPPH scavenging activity (55.25%), followed by umbelliferone)C**1**) (30.20%). Also, the two compounds showed DPPH antioxidant activity with IC_50_ values of 5.47 and 3.84 µg/mL for C1 and C2, respectively (Table 4).

**Table 4:** DPPH scavenging activity of compounds C1 and C2

Compounds	DPPH scavenging activity (%)	IC_50_ (µg/mL)
Umbelliferone (C1)	30.20	5.47
1-methoxy-3-methyl-8-hydroxy anthraquinone (C2)	55.25	3.84
Ascorbic acid	78.50	–

### In vitro DNA gyrase-B inhibition

**Fig. 3:**
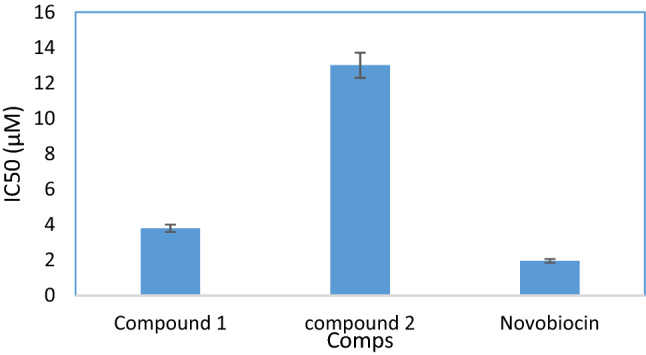
DNA Gyrase-B inhibition activity of the obtained compounds C1 and C2

DNA gyrase is a bacterial enzyme that catalyzes the ATP-dependent negative supercoiling of closed-circular double-stranded DNA. Gyrase is a member of the topoisomerase enzyme family, which is involved in the control of DNA topological transitions (Reece and Maxwell 1991). DNA gyrase is made up of two subunits: A and B. (Gyr-A and -B, respectively). Gyr-A is the component responsible for binding to DNA and relaxing its positive supercoils. Fluoroquinolone medicines are also effective against it. Gyr-B, on the other hand, is in charge of providing the necessary energy for this action by hydrolyzing one molecule of ATP (Zhuo et al. 2013). Accordingly, we tested the obtained purified compounds C1 and C2 for their Gyr-B inhibitory activity. As shown in Fig. 3, the two compounds showed inhibition of Gyr-B enzyme. Compound **C1** was the most potent inhibitor, with an IC_50_ value of (3.79 ± 0.21 µM), while compound **C2** was the least potent (IC_50_ = 13 ± 0.71 µM).

### The ADME-related physicochemical properties

**Fig. 4:**
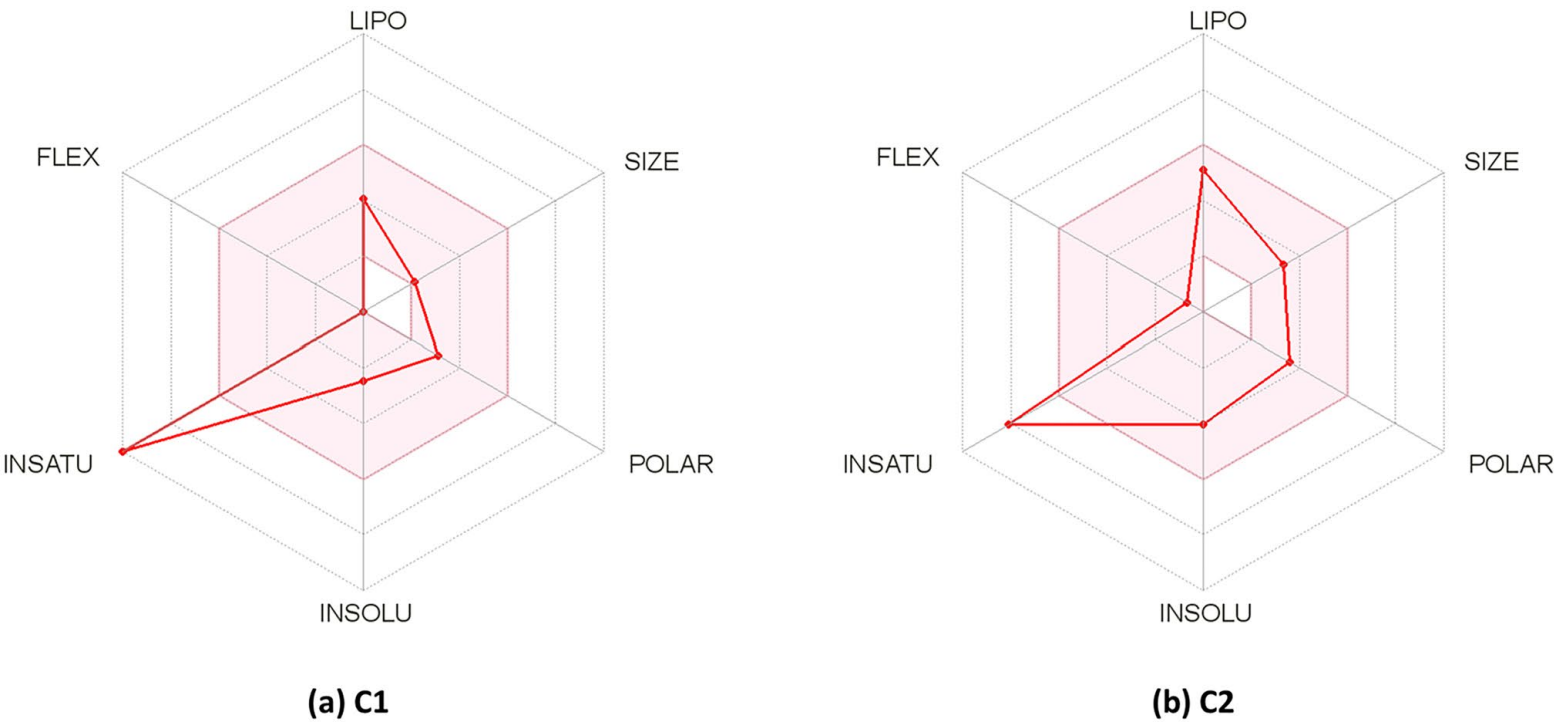
Bioavailability Radar plot of **a** **compound 1** and **b** **compound 2**. The pink area shows the optimal range for each property (Lipophilicity: XLOGP3 between − 0.7 and + 5.0, size: MW between 150 and 500 g/mol, polarity: TPSA between 20 and 130 Å^2^, solubility: log *S* not higher than 6, saturation: fraction of carbons in the sp^3^ hybridization not less than 0.25, and flexibility: no more than 9 rotatable bonds) (color figure online)

The ADME-related physicochemical properties of umbelliferone**) C1)** and 1-methoxy-3-methyl-8-hydroxy-anthraquinone **(C2)** ester were estimated using SwissADME online server (Daina et al. 2017). The measurements are based on the characterization of the purified compounds following the drug-likeness rules. Accordingly, the compound passed the Lipinski, Veber rules without any violation, while one violation (atoms < 20) has been detected with Ghose rule. On the other hand, **(C2)** passed the Lipinski, Veber, and Ghose rules filter with no violations. Both compounds exhibited 0.55% oral bioavailability and could be used as an oral medication (Table 5). Additionally, the rapid estimation of drug-likeness was performed by plotting of Bioavailability Radar plot of the two compounds which is based on six physicochemical parameters size, polarity, lipophilicity, solubility, flexibility, and saturation, while the pink area depicts the optimal value range for each parameter. According to the obtained diagram, the two compounds displayed optimal range (pink area) for all the parameters instauration parameter for **C1** and **C2** (Fig. 4a and b). Another important physicochemical parameter is Lipophilicity which shows the compound permeability across the cell membrane (Potts and Guy 1992; Rutkowska et al. 2013). Both tested compounds **(C1 and C2)** showed log *P*_o/w_ values below 5, (1.50 and 2.48) suggesting a good permeability and absorption across the cell membrane. Additionally, solubility is one of the most important parameters influencing compound absorption in any formulation process (Daina et al. 2017). Based on ESOL topological model, compound 1 is soluble, while compound **C2** is moderately soluble. For defining medicinal chemistry and Leadlikness, compound **C1** did not pass the rule of three (RO3), as it has one violation for this rule, while compound **2** passed the rule of three (RO3), with no violation. For synthetic accessibility score (SAscore) that estimated on the similarity of fragments and complexity penalties, both compounds **(C1 and C2)** showed moderate synthetic accessibility with values (2.56 and 2.57), respectively.

**Table 5:** ADME-related physicochemical parameters of dibutyl phthalate ester

Predictive models parameters		Compound 1	Compound 2
Physicochemical Properties	Molecular Weight	162.14	268.26
	Fraction Csp3	0.00	0.12
	Rotatable bonds	0	1
	H-bond acceptors	3	4
	H-bond donors	1	1
	Molar Refractivity	44.51	73.23
	Topological polar surface area (TPSA)	50.44 Å^2^	63.60 Å^2^
Lipophilicity	log *P*_o/w_ (XLOGP3)	1.58	3.40
	log *P*_o/w_ (WLOGP)	1.50	2.48
	log *P*_o/w_ (MLOGP)	1.04	1.17
Solubility	log *S* (ESOL)	− 2.46	− 4.02
	Solubility	5.66e–01 mg/mL; 3.49e–03 mol/L	2.54e–02 mg/mL; 9.48e–05 mol/L
	Class	Soluble	Moderately soluble
Druglikeness	Lipinski (RO5)	Yes; 0 violation	Yes; 0 violation
	Ghose	No; 1 violation: #atoms < 20	Yes
	Veber	Yes	Yes
	Bioavailability Score	0.55	0.55
Leadlikness	Rule of three (RO3)	No; 1 violation: MW < 250	Yes
	Synthetic accessibility	2.56	2.57
Pharmacokinetics Parameters	GI (HIA) absorption	High	High
	BBB permeant	Yes	Yes
	P-gp substrate	No	No
	CYP1A2 inhibitor	Yes	Yes
	CYP2C19 inhibitor	No	Yes
	CYP2C9 inhibitor	No	Yes
	CYP2D6 inhibitor	No	No
	CYP3A4 inhibitor	No	Yes
	log *K*_p_ (skin permeation: cm/s)	− 6.17 cm/s	− 5.52 cm/s

log *P*_o/w_ = The partition coefficient between *n*-octanol and water, log *S* = The decimal logarithm of the molar solubility in water. Lipinski (RO5) criteria range are lipophilicity (log *P*_o/w_) ≤ 5, MW ≤ 500, H-bond donors ≤ 5, and H-bond acceptors ≤ 10. Ghose filter criteria range is log *P*_o/w_ in − 0.4 to + 5.6 range, MR from 40 to 130, MW from 180 to 480, No. of atoms from 20 to 70. Veber rule criteria range are: RB ≤ 10 and TPSA ≤ 140 Å^2^. RO3 criteria range is XLOGP3 ≤ 3.5, MW ≤ 350, H-bond donors ≤ 3, H-bond acceptors ≤ 3, and RB ≤ 3. Synthetic accessibility (SA) score ranges from 1 (very easy) to 10 (very difficult)

*GI (HIA)* human gastrointestinal absorption, *BBB* Blood–brain barrier permeation, *P-gp* permeability glycoprotein, *log K*_*p*_ the skin permeability coefficient

**Fig. 5:**
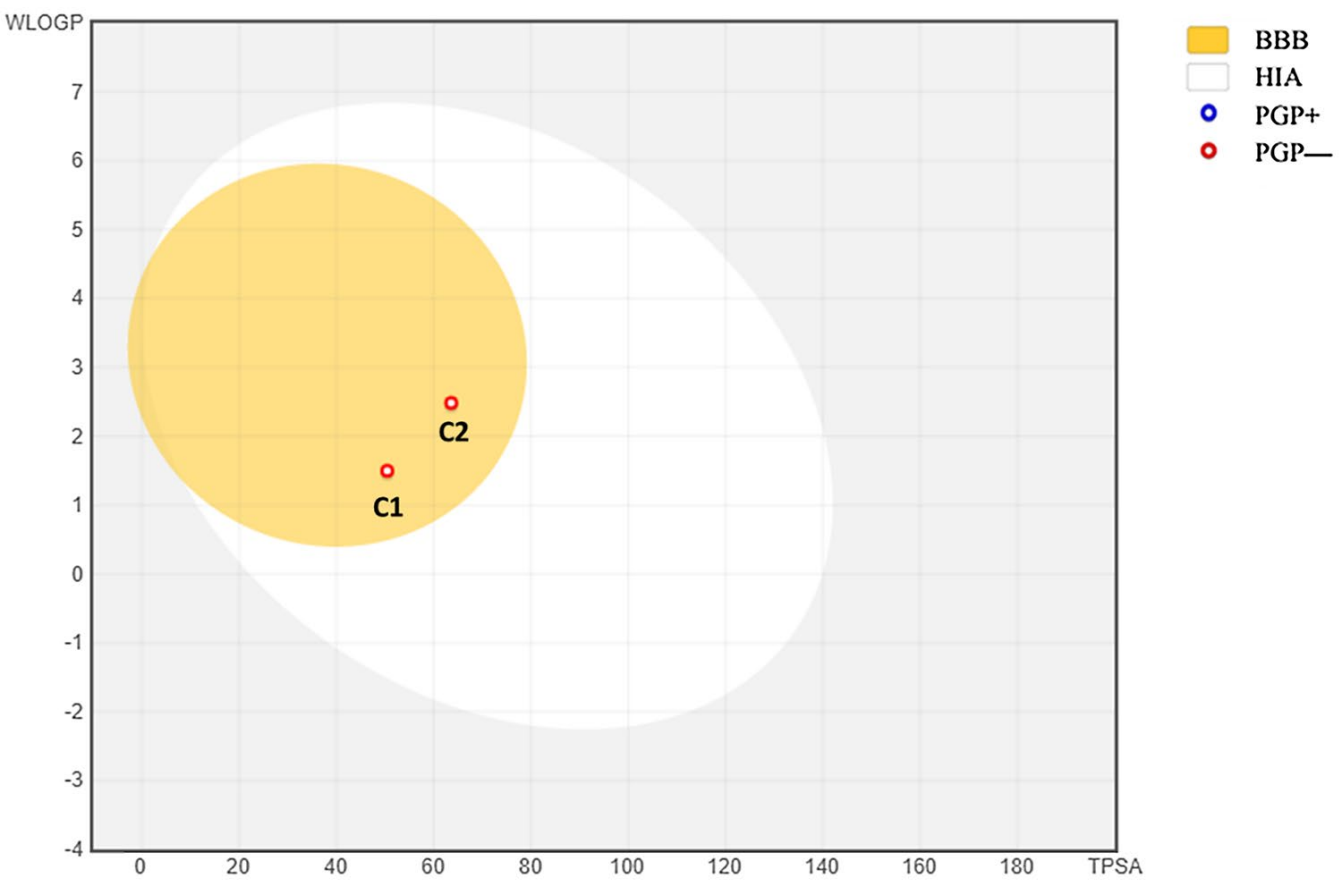
BOILED-Egg plot for both C1 and C2. The yellow zone (yolk) is for highly possible BBB permeability, while the white region (GI) is for highly probable HIA (GI) absorption. Molecules with minimal absorption and no brain penetration are represented by the outside gray zone. The points are also colored blue if P-gp substrate (PGP +) is expected, and red if P-gp non-substrate (PGP) is projected (color figure online)

Table 5, showed the compounds **(C1** and **C2)** pharmacokinetic parameters measured using the vector machine algorithm (SVM) model (Daina et al. 2017). Compound **C1** showed selective inhibitory activity toward CYP1A2 isoenzyme, while compound **C2** showed selective activity against CYP1A2, CYP2C19, and CYP2C9 isoenzymes. Figure 5 represents the BOILED-Egg model (Brain or Intestinal Estimate D permeation method, WLOGP vs TPSA) that was adapted from Daina et al (2017). The compounds **(C1** and **C2)** demonstrated high human gastrointestinal absorption (GI). The compounds are non-P-gp substrates (PGP-, red dots), while their blood–brain barrier (BBB) permeant (TPSA < 75 Å^2^), suggesting the presence of effects on the central nervous system (CNS) (Daina and Zoete 2016). Prediction of skin permeability coefficient (*K*_p_) of the obtained compound was done as described by Potts and Guy **(**Potts and Guy 1992). The two compounds **(C1** and **C2)** showed log (*K*p) (− 6.17 and − 5.52 cm/s), while the more negative log *K*_p_, the less skin permeant is the compounds.

### Toxicity prediction via ProTox ii

Toxicity prediction of the obtained compounds (**C1** and **C2)** was performed using ProTox ii webserver (Banerjee et al. 2018). Results in the Table 6 showed that compound **1** has no pronounced toxicity, while compound **2** showed activity toward some targets as predicted by ProTox ii.

**Table 6:** In silico toxicity prediction of purified compounds (C1 and C2)

Classification	Target	C1	C2
Organ toxicity	Hepatotoxicity	Inactive	Inactive
Toxicity end points	Carcinogenicity	Active	Inactive
	Immunotoxicity	Inactive	Active
	Mutagenicity	Inactive	Active
	Cytotoxicity	Inactive	Inactive
Tox21-Nuclear receptor signaling pathways	Aryl hydrocarbon Receptor (AhR)	Inactive	Active
	Androgen Receptor (AR)	Inactive	Inactive
	Androgen Receptor Ligand Binding Domain (AR-LBD)	Inactive	Inactive
	Aromatase	Inactive	Inactive
	Estrogen Receptor Alpha (ER)	Inactive	Active
	Estrogen Receptor Ligand Binding Domain (ER-LBD)	Inactive	Inactive
	Peroxisome Proliferator Activated Receptor Gamma (PPAR-Gamma)	Inactive	Inactive
Tox21-Stress response pathways	Nuclear factor (erythroid-derived 2)-like 2/antioxidant responsive element (nrf2/ARE)	Inactive	Inactive
	Heat shock factor response element (HSE)	Inactive	Inactive
	Mitochondrial Membrane Potential (MMP)	Inactive	Active
	Phosphoprotein (Tumor Supressor) p53	Active	Inactive
	ATPase family AAA domain-containing protein 5 (ATAD5)	Inactive	Inactive

## Conclusion

*Actinobacteria* are the most widely distributed microorganisms on earth with the ability to produce unique bioactive compounds with high commercial value. The strain ***Actinomyces* sp. AW6** showed pronounced antioxidant and antimicrobial potential against *E. coli*, *S. aureus*, *B. subtilis*, *P. aeruginosa*, *A. niger* and *C. albicans*. Cultivation of the selected strain led to the isolation of two compounds **C1**: umbelliferone and **C2:** 1-methoxy-3-methyl-8-hydroxy-anthraquinone. Both compounds displayed antibacterial activity toward *S. aureus* and *E. coli.* They also exhibited antioxidant activity and anti-Gyr-B enzyme activity with IC_50_ value of (3.79 ± 0.21 µM) for **C1**, and (IC_50_ = 13 ± 0.71 µM) for **C2**. The ADME-related physicochemical properties of the obtained compound were predicted using SwissADME web tools and the ProToxii webserver was used to estimate in silico toxicity.

## Supplementary Information

Below is the link to the electronic supplementary material.

Supplementary file1 (DOCX 1926 KB)

## Data Availability

Not applicable.
