# Supplementary material for: Bioactive secondary metabolites from marine Actinomyces sp. AW6 with an evaluation of ADME-related physicochemical properties
Source: Arch Microbiol. 2022 Aug 1;204(8):537. doi: 10.1007/s00203-022-03092-5 (PMC9343302; doi:10.1007/s00203-022-03092-5)
Supplement: Supplementary file 1 — Supplementary file1 (DOCX 1926 KB) [file 203_2022_3092_MOESM1_ESM.docx]

| **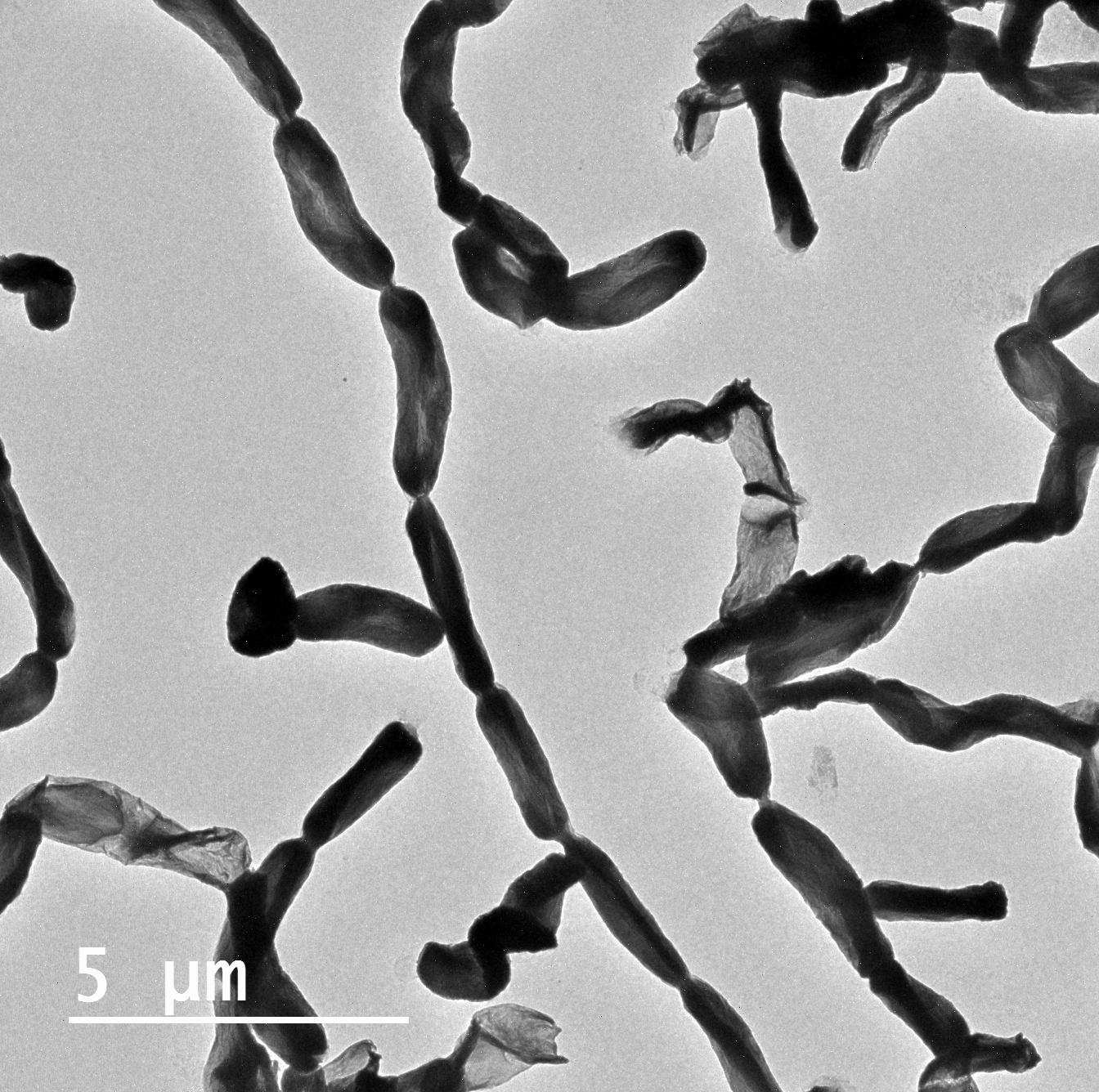** | *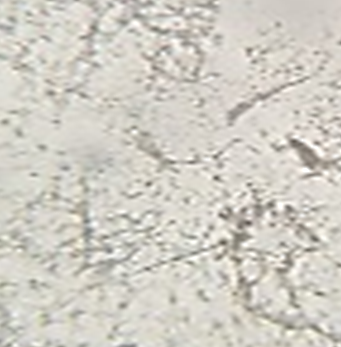* | *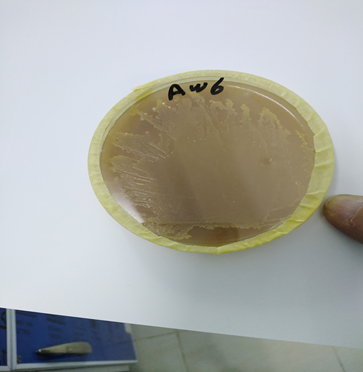* |
| --- | --- | --- |
| **Supplementary. 1** TEM photomicrograph showing rectiflexibiles spore chain and smooth surface (isolate **AW6**, × 8000)  **Supplementary. 2** Cultural properties of isolate no. AW6 on different cultural media   \| Medium \| Color of \| \| \| \| --- \| --- \| --- \| --- \| \| Aerial mycelium \| Diffusible pigments \| Substrate mycelium \| \| 1- Starch nitrate medium \| Yellow \| +ve \| Yellowish \| \| 2- Starch-ammonium sulphate medium \| Yellow \| +ve \| Yellowish-brown \| \| 3- Glycerol-asparagine medium \| Yellow \| +ve \| Grayish brown \| \| 4- Oat-meal medium \| Yellow \| +ve \| Yellowish-brown \| \| 5- yeast \ malt extract agar medium \| Pale brown \| +ve \| Pale brown \| \| 6- Czapeks medium \| brown \| +ve \| Yellowish brown \| | | |

**Supplementary. 3** Biochemical tests and carbon source utilization of isolate no. AW6

| **Strain** | **Biochemical tests** | | | | | | | | | | | **Carbon source utilization** | | | | | | | | | |
| --- | --- | --- | --- | --- | --- | --- | --- | --- | --- | --- | --- | --- | --- | --- | --- | --- | --- | --- | --- | --- | --- |
|  | **Voges-Proskauer** | **Oxidase** | **Indole** | **Alkaline phosphatase** | **Methyl red** | **Citrate** | **Nitrate** | **Urease** | **arginine dihydrolase** | **N-Acetyl-Glucosamine** | **Methyl-αD-Glucosamine-** | **Sucrose** | **Melibiose** | **Xylitol** | **Mannitol** | **Trehalose** | **Lactose** | **Maltose** | **Mannose** | **Fructose** | **Glucose** |
| **Aw6** | **-** | + | + | **+** | + | - | **+** | - | **+** | **-** | **-** | + | **-** | **-** | **+** | **+** | **+** | **+** | **+** | **+** | **+** |
